# The Distribution of Mobile Colistin-Resistant Genes, Carbapenemase-Encoding Genes, and Fluoroquinolone-Resistant Genes in *Escherichia coli* Isolated from Natural Water Sources in Upper Northeast Thailand

**DOI:** 10.3390/antibiotics11121760

**Published:** 2022-12-06

**Authors:** Pongthep Tabut, Rapeepan Yongyod, Ratchadaporn Ungcharoen, Anusak Kerdsin

**Affiliations:** The Faculty of Public Health, Kasetsart University Chalermphrakiat Sakon Nakhon Province Campus, Thailand. 59 Moo 1, Chiang Khruea Subdistrict, Mueang Sakon Nakhon District, Sakon Nakhon 47000, Thailand

**Keywords:** *mcr*, PMQR, carbapenemase, *Escherichia coli*, natural water, Namsuay watershed, antibiotic resistance, antimicrobial resistance

## Abstract

Antimicrobial resistance (AMR) is considered a serious problem in many countries, including Thailand. AMR and antibiotic resistance genes (ARGs) could transfer between humans, animals, and the environment causing a threat to human health. This study described the antibiotic resistance of *Escherichia coli* (*E. coli*) from surface water, wastewater, and discharge water in the Namsuay watershed in upper northeast Thailand. The water samples were collected in the dry and wet seasons. The 113 *E. coli* isolates were confirmed using a polymerase chain reaction and examined for their antibiotic susceptibility, ARGs, and genetic relationship. The results indicated that *E. coli* was resistant to the following classes of antibiotics: fluoroquinolone, third-generation cephalosporin, polymyxin, and carbapenem. The isolates carried the *mcr-1*, *mcr-8*, *mcr-9*, *bla*_oxa-48-like_, *aac(6′)-bl-cr*, *qepA*, and *oqxAB* genes. Phylogroup B1 was a predominant group among the *E. coli* in the study. In addition, the *E. coli* isolates from the discharge water (a hospital and a fish farm) had a higher prevalence of antibiotic resistance and harboured more ARGs than the other water sample sources. The presence of antibiotic-resistant *E. coli* and ARG contamination in the natural water source reflected an AMR management issue that could drive strategic policy regarding the active surveillance and prevention of AMR contamination.

## 1. Introduction

Antimicrobial resistance (AMR) is an increasing issue of global concern as it leads to antibiotic treatment failure that places a burden on public health, the economy, and the environment [1]. Human activities, such as agriculture, animal husbandry, and daily body hygiene, could cause wastewater, sewage contamination with chemicals, and faecal pollution, including causing antibiotics and antibiotic resistance genes (ARGs) to leak into the environment [2]. If wastewater management is not properly and effectively carried out, there may be adverse effects on the ecosystem and human health [3]. The identification of microorganisms in water is important to assess the safety and sanitation of water use from water sources. Among these organisms, *E. coli* is an indicator bacterium to measure the microbiological quality of water supplies. It is found in the intestines of humans and warm-blooded animals [4]. Furthermore, it is an important pathogen in both intra-intestinal tract and extra-intestinal tract infections in humans [5].

The inappropriate or overuse of antibiotics in both humans and animals is one action that could lead to AMR. Antibiotic contamination in the environment would encourage antibiotic-susceptible bacteria to become non-susceptible or resistant, as a consequence of selective pressures [6]. Mutations and evolution within selection due to antibiotic pressures or natural mechanism factors have resulted in the emergence of ARGs that could transfer antibiotic-resistance abilities to other bacteria via plasmids, transposons, chromosomal cassettes, and prophages [7]. ARGs, transferred by the mechanism of a horizontal gene, were found in bacteria from environmental samples, such as mobile colistin-resistant (*mcr*) genes [8,9], carbapenemase-encoding genes (CEG) [10], and plasmid-mediated-quinolone-resistance (PMQR) genes [11,12]. Therefore, areas with high use of antibiotics, such as hospitals, animal husbandries, and communities that do not control the use of antibiotics, are high-risk areas for AMR. These antibiotic resistance (AR) bacteria could contaminate water pollution sources, including hospital wastewater, community wastewater, livestock and fishery wastewater, and agricultural wastewater, by transmission mechanisms and many pathways. Humans acquire and emit AR bacteria through interactions between humans and the environment [13,14].

**Figure 1:**
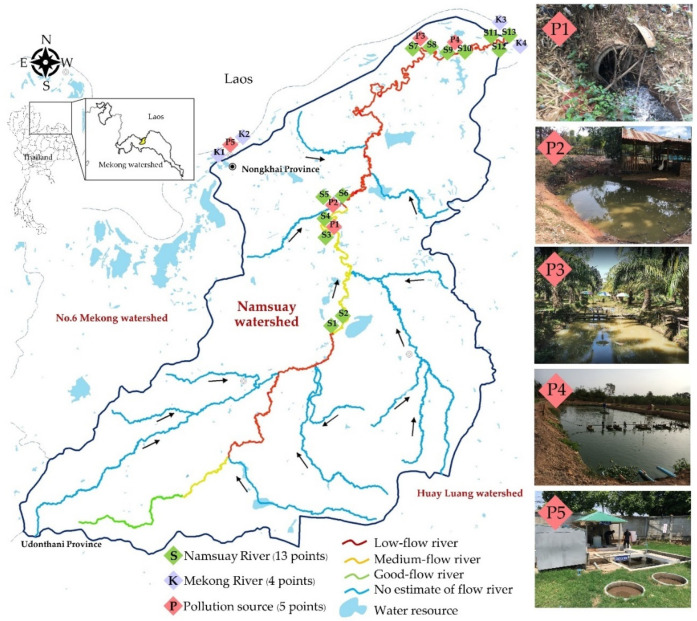
Study area and sampling sites. In total, 44 water samples were collected from 22 sites in the Nongkhai Province, northeast Thailand. The types of water sampling sites were: surface water (S1–S13 and K1–K4), wastewater (P1–P3), and discharge water (P4–P5).

Herein, we described the contamination of antibiotic-resistant *E. coli* carrying important ARGs, consisting of *mcr*, CEG, and PMQR genes, from a natural water source in upper northeast Thailand. The water sources consisted of surface water above the ground in the Namsuay River and Mekong River; wastewater in this study was defined as untreated, used water affected by domestic, agricultural, and poultry areas, whereas discharge water was defined as used treated water affected by hospitals and catfish farms, as shown the examples in Figure 1. This information could drive strategic policies for the active surveillance and prevention of AR bacterial contamination in natural water sources throughout the country.

## 2. Results

### 2.1. Microbiological Quality

As shown in Table 1, The most probable number (MPN) levels of the total coliforms and faecal coliforms in all samples were high. The surface water samples were highly contaminated with faecal coliform bacteria (43/44, 97.79%), except for the surface water samples from point K1 (Figure 1), which was less than 2 MPN/100 mL. The wastewater samples contained more faecal coliform bacteria than the other sources in the dry season (350 to >1600 MPN/100 mL) and the wet season (>1600 MPN/100 mL). It was clear that the faecal coliform bacteria count from the wastewater had a higher density than the other sampling types because the wastewater sources were untreated.

### 2.2. Distribution of the Antibiotic Resistance Gene Profiles of the E. coli Isolates

The current study focused on three types of ARGs of public health concern: the CEGs, *mcr*, and PMQR in 113 *E. coli* isolates from 44 water samples that consisted of surface water (*n* = 89), wastewater (*n* = 17), and discharge water (*n* = 7). The most common ARG patterns in *E. coli* were for *oqxAB* alone, followed by *mcr-9* alone, and *mcr-8*+*mcr-9*+ *bla*_oxa-48-like_. The *oqxAB* gene had a high prevalence in the wastewater and surface water samples, while the *mcr-9* gene had a high prevalence in only the surface water. The highest co-occurrences of the ARGs in *E. coli* were for *mcr-8*+*mcr-9*+ *bla*_oxa-48-like_ (Table 2).

As shown in Table 2, in the dry season, the prevalence levels of the ARGs were 81.25% (13/16) in the *E. coli* from the surface water samples and 66.67% (2/3) in the *E. coli* from the wastewater samples. A predominant ARG pattern in the *E. coli* was *mcr-9* and *mcr-8*+*mcr-9*+ *bla*_oxa-48-like_. In the wet season, the ARGs in the *E. coli* isolates from the wastewater samples (42.86%, 6/14) were higher than from the discharge water samples (40.00%, 2/5) and surface water samples (36.99%, 27/73). A predominant ARG pattern in *E. coli* was *oqxAB*.

### 2.3. Antibiotic Resistance Phenotypes of the E. coli Isolates

The antibiotic-resistant *E. coli* are shown in Table 2. *E. coli* from each water sample type had high resistance to ciprofloxacin, with 57.14% (4/7) in the discharge water samples, 29.41% (5/17) in the wastewater samples, and 16.85% (15/89) in the surface water samples.

In the dry season, the prevalence of antibiotic-resistant *E. coli* was detected in 100.00% of the wastewater (3/3) and discharge water (2/2) samples, while it was in 62.50% (10/16) of the surface water samples. The colistin resistance at 28.57% (6/21) was higher in *E. coli* compared to another antibiotic (Table 2).

In the wet season, the antibiotic resistance profiles of the *E. coli* isolates against the tested antibiotics in each water sample type were: discharge water, 60.00% (3/5), wastewater, 42.86% (6/14), and surface water, 23.39% (17/73). The most prevalent AR pattern was ciprofloxacin in 20.65% (19/92) (Table 2).

Table 3 shows the high distribution of antibiotic resistance in *E. coli* for ciprofloxacin (26.55%, 30/113), followed by colistin (7.96%, 9/113), cefotaxime (6.19%, 7/113), ceftazidime (3.54%, 4/113), and imipenem (3.54%, 4/113). The most prevalent ARG in antibiotic-resistant *E. coli* was *oqxAB* (7.08%, 8/113). In addition, all colistin-resistant *E. coli* harboured ARGs.

### 2.4. Phylogenetic Group of the E. coli Isolates

As shown in Table 4, the 113 *E. coli* isolates could be divided into phylogenetic groups, with 42.48% (43/113) in group B1, 10.62% (12/113) in group C, 9.73% (11/113) in groups A and E, 3.54% (4/113) in groups B2 and clade I or II, and 1.77% (2/113) in group F, while 18.58% (21/113) were unclassified into a phylogenetic group. The *E. coli* harbouring the *oqxAB* gene belonged to phylogroup B1 in this study. The *E. coli* harbouring co-ARGs were classified in phylogroups B1, B2, E, and clade I or II. Additionally, phylogroup B1 contained *E. coli*-harbouring ARGs (15.93%, 18/113) and antibiotic-resistant ones (7.08%, 8/113). However, the *oqxAB* gene was the most prevalent in phylogroup B1 (7.96%, 9/113). The *E. coli* resistance to ciprofloxacin demonstrated a relatively high prevalence in phylogroup C (5.31%, 6/113). In this study, the *E. coli* in phylogroup B2 (3.54%, 4/113) had a lower prevalence than the other phylogroups (A, B1, C, E, F, clade I or II, and unknown); however, all isolates harboured ARGs, as shown in Table 4.

### 2.5. Location of the Antibiotic-Resistant E. coli Isolates

**Figure 2:**
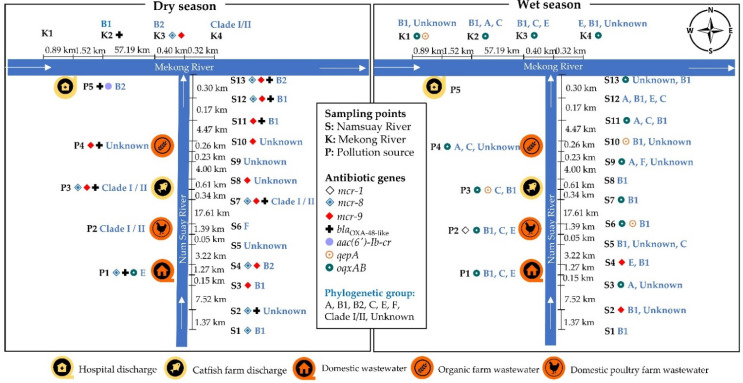
Distribution of the phylogenetic groups and antibiotic resistance genes, *mcr* (diamond symbol), CEGs (plus symbol) and PMQR (circle symbol) of *E. coli* at the water sample points.

The location of the antibiotic-resistant *E. coli* isolates was based on the water sampling points. Almost all sampling points had *E. coli* harbouring ARGs, except for two sampling points, namely the surface water from the Namsuay River (K1) in the dry season and the discharge water from a hospital (P5) in the wet season. Almost all water samples in the study were from agricultural areas, with a high prevalence of *mcr* genes in the dry season and a high prevalence of PMQR genes in the wet season, as shown in Figure 2.

In the dry season, *E. coli* was isolated from the water samples that carried the *mcr-8* gene from the surface water (S1, S2, S4, S7, S12, S13, and K3), domestic wastewater (P1), and fish farm discharge (P3), the *mcr-9* gene from surface water (S3, S4, S7, S8, S10, S11, S12, S13, and K3), fish farm (P3), and agricultural wastewater (P4), the *bla*_oxa-48-like_ gene from surface water (S2, S7, S11, S12, S13, and K2), domestic wastewater (P1), fish farm discharge (P3), agricultural wastewater (P4), and hospital discharge (P5), the *aac(6′)-bl-cr* gene from hospital discharge (P5), and the *oqxAB* gene from domestic wastewater (P1).

In the wet season, *E. coli* carried the *mcr-1* gene from a poultry farm (P2), the *mcr-9* gene from surface water (S2 and S4), the *qepA* gene from surface water (S6, S10, and K1) and fish farm discharge (P3), and the *oqxAB* gene from surface water (S3, S6, S7, S9, S11, S13, K1, K2, K3, and K4), domestic wastewater (P1), poultry farm (P2), fish farm discharge (P3), and agricultural wastewater (P4).

The diversity of the phylogenetic groups of *E. coli* indicated that there were several groups in each of the water sample types or each water sampling point. The *E. coli* strains belonging to phylogenetic group B2 were only found in the water samples from the dry season, and all isolates harboured ARGs. The B2 phylogenetic group samples harbouring the ARGs isolates had the *mcr-8* and *mcr-9* genes at S4 (the sampling point of the surface water of the Namsuay River near the untreated domestic wastewater), the *mcr-8*, *mcr-9*, and *bla*_oxa-48-like_ genes at S13 (the sampling point at the river mouth of the Namsuay River), the *mcr-8* and *mcr-9* genes at K3 (the sampling point at the surface of the Mekong River), and the *bla*_oxa-48-like_ and *aac(6′)-bl-cr* genes at P5 (the sampling point for the hospital discharge water).

### 2.6. Associations of Phenotypic and Genotypic Antibiotic Resistance in E. coli with the Phylogenetic Groups and Seasons

As shown in Table 5, Fisher’s exact test showed a significant association between the antibiotic resistance phenotype of *E. coli* and phylogroup non-B1 (*p* < 0.001). In contrast, there was no association between the resistance genes and phylogroups.

In the case of the season, the antibiotic resistance and the resistance genes of *E. coli* were significantly different between seasons (*p* < 0.001; Table 6).

## 3. Discussion

This study investigated the numerous ARG- and AR-*E. coli* isolates found in the Namsuay watershed sources. Several reports have indicated that bacterial isolates carry many ARGs in aquatic environments in Thailand [15,16,17]. One study revealed that the AR bacteria and ARGs in aquatic settings could be harmful to human health [18].

According to the current results, most of the *E. coli* isolates were resistant to ciprofloxacin, which belongs to the fluoroquinolone class and was found in all settings. The global antimicrobial resistance surveillance system (GLASS) reported the prevalence of resistance of *E. coli* from patients in Thailand to fluoroquinolone (54%), third-generation cephalosporin (38%), polymyxin (13%), and carbapenems (2%) [19]. The current study identified high fluoroquinolone-nonsusceptible *E. coli*, perhaps due to the higher levels of quinolone used in agriculture or communities that may transmit to humans via the food-chain system [20].

The prevalence levels of the ARGs in *E. coli* from the discharge water and wastewater were higher than for the surface water because the discharge water (a hospital and a fish farm) and the wastewater are anthropogenic sources that have a well-known origin of contributing to the spread of antibiotic resistance in the environment [21,22,23]. The most prevalent PMQR gene was *oqxAB*, which was consistent with the results of a study from China [24] and Thailand [25]. The *oqxAB* was more prevalent in the wet season than in the dry season. We carried the assumption that the dry season had less rainfall than the wet season because the runoff associated with rainfall was a driver of AR gene dissemination and contamination in water [26]. The most prevalent *mcr* gene was *mcr-9*. Other studies reported *mcr-9* in *E. coli* from animals in China and the environment in Germany [9]. In Thailand, *mcr-9* was detected in *Enterobacter cloacae* from patients with community-acquired urinary tract infections [27] and in *E. coli* from slaughtered pigs [28], indicating that *mcr-9* had spread in the environment and was circulating in the human-animal-environment.

The levels of diversity of the ARGs and antibiotic resistance in the dry and wet seasons were significantly different. Similarly, another study reported that the ARGs of E. coli in summer were more diverse than in spring, fall, and winter because of environmental factors, such as temperature, suited to the survival of bacteria [29]. A study in Bangladesh showed that the carbapenem-resistant E. coli from river water samples only had a higher prevalence level in summer compared to winter and that seasonal factors were not positively correlated in any other water systems [30]. The possible reasons for the seasonal variation were the differences in the temperature, pH, and electrical conductivity of the water. The dry season has less rainfall than the wet season, thus reducing the flow of water in rivers and causing a greater accumulation of bacteria in the water sources than in the wet season [26]. The runoff and leaching of rainwater could dissolve environmental contaminants as a part of natural recovery. In addition, the temperatures in the dry season are higher than in the wet season, allowing bacteria to develop better and resulting in a greater likelihood of finding bacteria resistant to antibiotics in the dry season.

Our study demonstrated that the phylogroup B1 was predominant, and its occurrence was generally commensal with faecal flora *E. coli* strains [31,32]. In Kuwait, most *E. coli* from sewage belonged to groups A and B1 [33]. Another study showed that pathogenic *E. coli* isolates were mostly in phylogroup B2, while the isolates from faecal flora were mostly in phylogroup B1 [32]. Although phylogenetic group B1 was related to commensal strains, it carried more ARGs and AR than the other groups. This might provide a reservoir for the spread or transmission of ARGs to other bacteria, as well as provide a human-animal-environmental interface.

## 4. Materials and Methods

### 4.1. Study Area and Sampling Sites

The study area for sampling was the Namsuay watershed (1321.91 km^2^) within the northeast Mekong watershed. The Namsuay River originates in the Udonthani province, from where it flows into the Mekong River in the Nongkhai province (Figure 1). The sample sites were selected to represent the different land uses and critical sites, such as agricultural, residential, cattle, and recreational sites.

In total, 44 water samples from 22 sites in the Nongkhai province (Figure 1) were collected from the Namsuay River surface water (*n* = 26), Mekong river surface water (*n* = 8), wastewater (*n* = 6), and discharge water (*n* = 4). At each sampling point, approximately 400 mL of water was collected in a sterile 500 mL glass bottle. Na_2_S_2_O_3_ was added for de-chlorination, and all samples were transported to the laboratory in a cold box within 24 h. The water samples from each site were collected seasonally in January 2021 during the dry season and in May 2021 during the wet season.

### 4.2. Microbiological Quality Assessment

The MPN analysis was performed to determine the total coliform bacteria and faecal coliform bacteria in the water samples using the American public health association method [4]. Metallic green sheen colonies from the completed phase of the MPN test in eosin methylene blue agar were selected to culture on trypticase soy agar to confirm the *E. coli* isolates using a polymerase chain reaction (PCR) as described elsewhere [34].

### 4.3. Detection of the Antibioic Resistance Genes in the E. coli Isolates

Mobile-colistin-resistant genes, *mcr-1* to *mcr-9*, were detected based on the PCR as described previously [28], and all *mcr* PCR products were subjected to Sanger sequencing for confirmation by Apical Scientific Sdn Bhd (Selangor, Malaysia). The CEGs (*bla*_IMP_, *bla*_KPC_, *bla*_NDM_, and *bla*_OXA-48-like_) were identified using multiplex PCR described elsewhere [35]. The PMQR genes (*qnrA*, *qnrB*, *qnrC*, *qnrS*, *aac(6′)-Ib* and *qepA*) were identified using multiplex PCR as described by [36].

### 4.4. Antibiotic Susceptibility Profiles of the E. coli Isolates

The antibiotic susceptibility testing was performed using disk diffusion and broth microdilution (colistin only). Both methods were carried out in accordance with the 2020 Clinical and Laboratory Standards Institute (CLSI) guidelines [37]. *E. coli* strain ATCC 25922^TM^ was used as the control. The four antibiotic classes selected for the disk diffusion assay were fluoroquinolones: ciprofloxacin (CIP, 5 µg), third-generation cephalosporins: cefotaxime (CTX, 30 µg) and ceftazidime (CTZ, 30 µg), and the carbapenem: imipenem (IMI, 10 µg). The minimum inhibitory concentration (MIC) for colistin in the polymyxins class of antibiotics was performed using the broth microdilution method at concentrations of 1, 2, 4, 8, 16, and 32 µg/mL. The results were interpreted according to the CLSI [37], with a colistin MIC of >4 µg/mL against *E. coli*, corresponding to resistance.

### 4.5. E. coli Phylogenetic Group

Clermont PCR typing was applied to classily all *E. coli* isolates into phylogroups A, B1, B2, C, D, E, F, clade I, or clade II, as described elsewhere [38].

### 4.6. Statistical Analysis

Fisher’s exact test (two-tailed) [39] was applied to find the associations between the antibiotic resistance phenotypes and antibiotic-resistant genes of the E. coli isolates and the phylogenetic groups (B1 and non-B1) and the seasons (dry and wet).

## 5. Conclusions

This study described the antibiotic resistance phenotypes and genotypes of *E. coli* in the Namsuay watershed, northeast rural Thailand. The results indicated that *E. coli* was resistant to the following classes of antibiotics: fluoroquinolone, third-generation cephalosporin, polymyxin, and carbapenem. The *E. coli* isolates carried the antibiotic resistance genes *mcr-1*, *mcr-8*, *mcr-9*, *bla*_oxa-48-like_, *aac(6′)-bl-cr*, *qepA,* and *oqxAB*. Furthermore, this study showed that there was a significant association between antibiotic resistance and the antibiotic-resistance genes of *E. coli* isolates and seasons. The *E. coli* isolates from discharge water (from a hospital and a fish farm) showed a prevalence of resistance to antibiotics and harboured ARGs at higher levels than the other water sample sources. The presence of antibiotic-resistant *E. coli* in surface water, wastewater, and discharge water provided evidence that there is a public health risk associated with human exposure to water such as the Namsuay watershed.

## Figures and Tables

**Table 1:** Total coliform bacteria and faecal coliform bacteria.

Season	Sample Type	Number of Samples	Total Coliform Bacteria (MPN/100 mL, Number of Samples)		Faecal Coliform Bacteria (MPN/100 mL, Number of Samples)	
			**Maximum (N)**	**Minimum (N)**	**Maximum (N)**	**Minimum (N)**
Dry	Surface water	17	>1600.00, (1)	22.00, (1)	350.00, (1)	2.00, (2)
	Wastewater	3	>1600.00, (3)	-	>1600.00, (2)	350.00, (1)
	Discharge water	2	>1600.00, (2)	-	>1600.00, (1)	79.00, (1)
Wet	Surface water	17	>1600.00, (7)	41.00, (1)	>1600.00, (4)	79.00, (4)
	Wastewater	3	>1600.00, (2)	350.00, (1)	>1600.00, (3)	-
	Discharge water	2	>1600.00, (1)	79.00, (1)	>1600.00, (1)	0.00, (1)

**Table 2:** Prevalence of the genotypic and phenotypic patterns of the *E. coli* isolates from each water source during the dry and wet seasons.

Genotypic and Phenotypic Patterns	Dry Season (*n* = 21)			Wet Season (*n* = 92)			Total (*n* = 113)		
	Surface Water (*n* = 16)	Wastewater (*n* = 3)	Discharge Water (*n* = 2)	Surface Water (*n* = 73)	Wastewater (*n* = 14)	Discharge Water (*n* = 5)	Surface Water (*n* = 89)	Wastewater (*n* = 17)	Discharge Water (*n* = 7)
ARG	13 (81.25)	2 (66.67)	2 (100.00)	27 (36.99)	6 (42.86)	2 (40.00)	40 (44.94)	8 (47.06)	4 (57.14)
*mcr-8*	1 (6.25)						1 (1.12)		
*mcr-9*	4 (25.00)			4 (5.48)			8 (8.99)		
*oqxAB*				20 (27.4)	5 (35.71)	1 (20.00)	20 (22.47)	5 (29.41)	1 (14.29)
*qepA*				1 (1.37)		1 (20.00)	1 (1.12)		1 (14.29)
*bla*_oxa-48-like_	1 (6.25)						1 (1.12)		
*mcr-8,mcr-9*	2 (12.5)						2 (2.25)		
*oqxAB, qepA*				2 (2.74)			2 (2.25)		
*mcr-1, oqxAB*					1 (7.14)			1 (5.88)	
*mcr-8,bla*_oxa-48-like_	1 (6.25)						1 (1.12)		
*mcr-9, bla*_oxa-48-like_	1 (6.25)	1 (33.33)					1 (1.12)	1 (5.88)	
*aac(6′)-Ib-cr, bla*_oxa-48-like_			1 (50.00)						1 (14.29)
*mcr-8,mcr-9, bla*_oxa-48-like_	3 (18.75)		1 (50.00)				3 (3.37)		1 (14.29)
*mcr-9, oqxAB, bla*_oxa-48-like_		1 (33.33)						1 (5.88)	
Not detect ARGs	3 (18.75)	1 (33.33)		46 (63.01)	8 (57.14)	3 (60)	49 (55.06)	9 (52.94)	3 (42.86)
AR	10 (62.50)	3 (100.00)	2 (100.00)	17 (23.29)	6 (42.86)	3 (60.00)	27 (30.34)	9 (52.94)	5 (71.43)
CIP	3 (18.75)	1 (33.33)	1 (50.00)	12 (16.44)	4 (28.57)	3 (60.00)	15 (16.85)	5 (29.41)	4 (57.14)
COL	5 (31.25)	1 (33.33)					5 (5.62)	1 (5.88)	
IMP				2 (2.74)			2 (2.25)		
CTX + COL	1 (6.25)						1 (1.12)		
IMP + COL	1 (6.25)						1 (1.12)		
CIP + COL		1 (33.33)						1 (5.88)	
CIP + CTX				1 (1.37)			1 (1.12)		
IMP + CTX				1 (1.37)			1 (1.12)		
CIP + CTZ + CTX			1 (50.00)	1 (1.37)	2 (14.29)		1 (1.12)	2 (11.76)	1 (14.29)
Susceptibility	6 (37.5)			56 (76.71)	8 (57.14)	2 (40)	62 (69.66)	8 (47.06)	2 (28.57)

ARG, antibiotic resistance gene; AR, antibiotic resistance; CIP, ciprofloxacin; COL, colistin; IMP, imipenem; CTX, cefotaxime; CTZ, ceftazidime.

**Table 3:** Relationships between the antibiotic resistance of the *E. coli* isolates (*n* = 113) and the resistance genes.

Antibiotic Resistance Gene Patterns	Fluoroquinolone	Carbapenem	3rd Generation of Cephalosporins		Polymyxin
	Ciprofloxacin	Imipenem	Ceftazidime	Cefotaxime	Colistin
ARGs not detected	15 (13.27)	2 (1.77)	2 (1.77)	4 (3.54)	
*mcr-8*					1 (0.88)
*mcr-9*					3 (2.65)
*oqxAB*	8 (7.08)		1 (0.88)	1 (0.88)	
*qepA*	1 (0.88)	1 (0.88)			
*bla*_oxa-48-like_	1 (0.88)				
*mcr-8 + mcr-9*					1 (0.88)
*oqxAB + qepA*	1 (0.88)				
*mcr-1+ oqxAB*	1 (0.88)				
*mcr-8+ bla*_oxa-48-like_					
*mcr-9+ bla*_oxa-48-like_					1 (0.88)
*aac(6′)-Ib-cr + bla*_oxa-48-like_	1 (0.88)		1 (0.88)	1 (0.88)	
*mcr-8 + mcr-9 + bla*_oxa-48-like_	1 (0.88)	1 (0.88)		1 (0.88)	2 (1.77)
*mcr-9 + oqxAB + bla*_oxa-48-like_	1 (0.88)				1 (0.88)
Total	30 (26.55)	4 (3.54)	4 (3.54)	7 (6.19)	9 (7.96)

ARGs, antibiotic resistance genes.

**Table 4:** Profiles of the antibiotic resistance genes and the antibiotic resistance of *E. coli* according to the phylogenetic group.

Profile	A	B1	B2	C	E	F	Clade I or II	Unknown	Total
Number of isolates	11 (9.73)	48 (42.48)	4 (3.54)	12 (10.62)	4 (3.54)	11 (9.73)	2 (1.77)	21 (18.58)	113 (100.00)
Antibiotic resistance genes	5 (4.42)	18 (15.93)	4 (3.54)	5 (4.42)	2 (1.77)	8 (7.08)	1 (0.88)	9 (7.96)	52 (46.02)
*mcr-8*		1 (0.88)							1 (0.88)
*mcr-9*		3 (2.65)			2 (1.77)			3 (2.65)	8 (7.08)
*oqxAB*	4 (3.54)	9 (7.96)		4 (3.54)	4 (3.54)	1 (0.88)		4 (3.54)	26 (23.01)
*qepA*	1 (0.88)			1 (0.88)					2 (1.77)
*bla_oxa-48-like_*		1 (0.88)							1 (0.88)
*mcr-8 + mcr-9*			2 (1.77)						2 (1.77)
*oqxAB + qepA*		2 (1.77)							2 (1.77)
*mcr-1 + oqxAB*					1 (0.88)				1 (0.88)
*mcr-8 + bla_oxa-48-like_*								1 (0.88)	1 (0.88)
*mcr-9 + bla_oxa-48-like_*		1 (0.88)						1 (0.88)	2 (1.77)
*aac(6′)-Ib-cr + bla_oxa-48-like_*			1 (0.88)						1 (0.88)
*mcr-8 + mcr-9 + bla_oxa-48-like_*		1 (0.88)	1 (0.88)				2 (1.77)		4 (3.54)
*mcr-9 + oqxAB + bla_oxa-48-like_*					1 (0.88)				1 (0.88)
Undetectable ARGs	6 (5.31)	30 (26.55)		7 (6.19)	3 (2.65)	1 (0.88)	2 (1.77)	12 (10.62)	61 (53.98)
Antibiotic resistance	3 (2.65)	8 (7.08)	3 (2.65)	6 (5.31)	4 (3.54)	6 (5.31)	1 (0.88)	10 (8.85)	41 (36.28)
CIP	1 (0.88)	4 (3.54)		6 (5.31)	3 (2.65)	5 (4.42)	1 (0.88)	4 (3.54)	24 (21.24)
COL		2 (1.77)	1 (0.88)					3 (2.65)	6 (5.31)
IMP	2 (1.77)								2 (1.77)
CTX + COL					1 (0.88)				1 (0.88)
IMP + COL			1 (0.88)						1 (0.88)
CIP + COL						1 (0.88)			1 (0.88)
CIP + CTX								1 (0.88)	1 (0.88)
IMP + CTX		1 (0.88)							1 (0.88)
CIP + CTZ + CTX		1 (0.88)	1 (0.88)					2 (1.77)	4 (3.54)
Susceptibility	8 (7.08)	40 (35.4)	1 (0.88)	6 (5.31)		5 (4.42)	1 (0.88)	11 (9.73)	72 (63.72)

ARGs, antibiotic resistance genes; AR, antibiotic resistance; CIP, ciprofloxacin; COL, colistin; IMP, imipenem; CTX, cefotaxime; CTZ, ceftazidime.

**Table 5:** Associations between the antibiotic susceptibility and antibiotic resistance genes of *E. coli* and Phylogenetic Group (B1 and Non-B1).

Phenotypic and Genotypic Antibiotic Resistance in *E. coli*	B1 (*n* = 48)	Non-B1 (*n* = 65)	*p*-Values
Antibiotic resistance	8	33	<0.001
Antibiotic susceptibility	40	32	
Antibiotic resistance genes	18	34	0.131
Non-antibiotic resistance genes	30	31	

**Table 6:** Association between antibiotic susceptibility and antibiotic resistance genes of *E. coli* and season (dry and wet).

Phenotypic and Genotypic Antibiotic Resistance in *E. coli*	Dry (*n* = 21)	Wet (*n* = 92)	*p*-Values
Antibiotic resistance	15	26	<0.001
Antibiotic susceptibility	6	66	
Antibiotic resistance genes	17	35	<0.001
Non-antibiotic resistance genes	4	57	

## Data Availability

Not applicable.
